# Microstructural Changes in the Corpus Callosum in Systemic Lupus Erythematous

**DOI:** 10.3390/cells12030355

**Published:** 2023-01-18

**Authors:** Paulo Rogério Julio, Thais Caldeira, Gustavo Retuci Pinheiro, Carla Helena Capello, Renan Bazuco Fritolli, Roberto Marini, Fernando Cendes, Paula Teixeira Fernandes, Lilian T. L. Costallat, Leticia Rittner, Simone Appenzeller

**Affiliations:** 1 Graduate Student at Child and Adolescent Health Program, School of Medical Science, University of Campinas, Campinas 13083-970, SP, Brazil; 2 Autoimmunity Laboratory, School of Medical Science, University of Campinas, Campinas 13083-970, SP, Brazil; 3 Medical Image Computing Laboratory, School of Electrical and Computer Engineering, University of Campinas (UNICAMP), Campinas 13083-852, SP, Brazil; 4 Graduate Program Physiopathology Program, School of Medical Science, University of Campinas, Campinas 13083-970, SP, Brazil; 5 Department of Pediatrics, School of Medical Science, University of Campinas, Campinas 13083-970, SP, Brazil; 6 Department of Neurology, School of Medical Sciences, University of Campinas (UNICAMP), Campinas 13083-970, SP, Brazil; 7 Department of Sport Sciences, Faculty of Physical Education, University of Campinas, Campinas 13083-970, SP, Brazil; 8 Rheumatology Unit, Department of Orthopedics, Rheumatology and Traumatology, School of Medical Sciences, University of Campinas (UNICAMP), Campinas 13083-970, SP, Brazil

**Keywords:** cSLE, aSLE, corpus callosum, atrophy

## Abstract

Central nervous system (CNS) involvement in childhood-onset systemic lupus erythematosus (cSLE) occurs in more than 50% of patients. Structural magnetic resonance imaging (MRI) has identified global cerebral atrophy, as well as the involvement of the corpus callosum and hippocampus, which is associated with cognitive impairment. In this cross-sectional study we included 71 cSLE (mean age 24.7 years (SD 4.6) patients and a disease duration of 11.8 years (SD 4.8) and two control groups: (1) 49 adult-onset SLE (aSLE) patients (mean age of 33.2 (SD 3.7) with a similar disease duration and (2) 58 healthy control patients (mean age of 29.9 years (DP 4.1)) of a similar age. All of the individuals were evaluated on the day of the MRI scan (Phillips 3T scanner). We reviewed medical charts to obtain the clinical and immunological features and treatment history of the SLE patients. Segmentation of the corpus callosum was performed through an automated segmentation method. Patients with cSLE had a similar mid-sagittal area of the corpus callosum in comparison to the aSLE patients. When compared to the control groups, cSLE and aSLE had a significant reduction in the mid-sagittal area in the posterior region of the corpus callosum. We observed significantly lower FA values and significantly higher MD, RD, and AD values in the total area of the corpus callosum and in the parcels B, C, D, and E in cSLE patients when compared to the aSLE patients. Low complement, the presence of anticardiolipin antibodies, and cognitive impairment were associated with microstructural changes. In conclusion, we observed greater microstructural changes in the corpus callosum in adults with cSLE when compared to those with aSLE. Longitudinal studies are necessary to follow these changes, however they may explain the worse cognitive function and disability observed in adults with cSLE when compared to aSLE.

## 1. Introduction

Childhood-onset systemic lupus erythematosus (cSLE) is a complex, inflammatory autoimmune disease characterized by the production of autoantibodies, with the involvement of several organs and tissues [1,2,3,4]. Compared to adult-onset (aSLE), cSLE patients generally have a more severe disease and more frequent neuropsychiatric manifestations [5,6]. Long-term disability has been described, including cognitive impairment in different domains, leading to difficulties in academic performance and employment [7,8,9].

Magnetic resonance imaging (MRI) has shown regional and diffuse brain atrophy, white matter (WM) lesions, and functional changes in SLE, independently of the age of disease onset. Disease activity, disease duration, autoantibodies, corticosteroid use, and CNS manifestations are associated with structural brain abnormalities [3,10,11].

The corpus callosum is the main axonal commissure formed by dense myelinated fibers, which interconnect the homologous territories of the two cerebral hemispheres [12,13,14,15]. Several pathologies including those of inflammatory, vascular, and autoimmune origin may affect the corpus callosum in a peculiar way, either in isolation or in association with other brain alterations. Recent studies have shown atrophy in a significant number of patients with cSLE, which is associated with low complement levels and an acute confusional state [16].

Diffusion tensor is an advanced MRI technique that is based on measurements of water diffusion in cellular compartments [3,17]. Water diffusion depends on orientation, spacing, and structural barriers, such as myelin and cellular membranes, in the brain tissue [18,19]. By analyzing the structure and integrity, DTI shows microstructural abnormalities that are not visualized by traditional MRI techniques. In SLE, DTI has shown lower fractional anisotropy (FA) and increased mean diffusivity (MD) in different brain structures, especially in SLE patients with neuropsychiatric manifestations [19]. However, the majority of studies focused on identifying microstructural abnormalities in SLE with neuropsychiatric manifestations when compared to SLE patients without these manifestations or healthy controls. More recently, our group has shown that DTI-based scalar maps allow parcellation of the corpus callosum in five different areas, corresponding to fibers received from different brain regions [20]. Since different cognitive functions are impaired in SLE, we hypothesized that the corpus callosum is not uniformly affected.

Therefore, the objective of this study was to analyze the microstructural abnormalities of the corpus callosum in adults with SLE with disease onset during childhood (cSLE) and during adulthood (aSLE) with similar disease durations. In addition, we analyzed the different areas of the corpus callosum to determine which areas are most affected by clinical, immunological, and treatment features and different durations of the disease.

## 2. Materials and Methods

### 2.1. Patients

We included consecutive SLE patients followed regularly at the pediatric and adult rheumatology outpatient clinic of the University of Campinas between October 2017 and December 2019. For inclusion in the present study, SLE patients had to: (1) fulfill the American College of Rheumatology (ACR) criteria for SLE [21,22] and the ACR/European League Against Rheumatism (EULAR) [23]; (2) be under 18 years of age at disease onset for the cSLE group and over the age of 18 for aSLE group, and (3) have a minimum follow-up period of 6 months. Patients that were unable to undergo MRI (claustrophobia (3 patient) and braces (10 patients)) and patients with CNS manifestations unrelated to SLE were excluded.

### 2.2. Controls

We invited the friends (without a personal or family history of autoimmune disease) of the SLE patients to be part of the control groups to assure similar sex and socio-demographic backgrounds. Control subjects with a prior history of a psychiatric disorder diagnosed by a medical practitioner, or those who had received psychotropic medications, were excluded (3 controls).

### 2.3. Ethics Approval

This study was approved by the local ethics committee (CAAE 72889717.8.0000.5404). All patients and controls (or legal guardians of minors) provided written informed consent.

### 2.4. Clinical Evaluation

A computer database was used to store the clinical and serologic characteristics of each patient. The following variables were included in this database: onset of the disease (age at which the first symptoms clearly attributable to SLE occurred); age at diagnosis (age at which the patients fulfilled 4 or more of the 1982 revised criteria for the classification of SLE [21]); and follow-up time (time from disease onset until date of MRI). Clinical and laboratory manifestations retrieved from medical charts were defined according to current guidelines [21,24,25].

### 2.5. Disease Activity/Cumulative Damage Evaluation

The Systemic Lupus Erythematous Disease Activity Index (SLEDAI) was used to define disease activity and a score of ≥3 was considered to represent active disease [26,27]. The systemic Lupus International Collaborating Clinics/ACR Damage Index (SDI) was used to define damage [28].

### 2.6. Neuropsychiatric (NP) Evaluation

All patients and controls underwent complete NP evaluation [29,30]. NP manifestations were categorized as being present at disease onset (≤6 months of disease) or during the follow-up period (>6 months of disease).

The instrument used to screen for cognitive impairment was the Montreal Cognitive Assessment (MoCa) in the translated version and validated for the Brazilian population [31]. The MoCa instrument evaluates eight domains, with scores from 0 to 30 points, where the higher the score, the better the cognitive function of the individual. The score is divided into short-term memory (5 points), visuospatial skills (4 points), executive function (4 points), attention, concentration and working memory (6 points), language (5 points), orienteering time (3 points), and space orientation (3 points) [31]. Beck Depression Inventory (BDI) [32,33] and Beck Anxiety Inventory (BAI) were applied to each individual [34]. 

### 2.7. MRI Acquisition

All of the subjects underwent MRI examination with a Philips 3T scanner. Sagittal T1-weighted images (3-dimensional acquisitions obtained in the sagittal T1-weighted gradient echo plane with 1 mm thickness, an angle of excitation of 35, a repetition time of 22 ms, a time to echo of 9 ms, a matrix of 240 × 240 pixels, a field of view of 230 × 250 cm, 1 × 1 pixel).

Diffusion weighted images (DWI) were acquired in the same scan with a 1 × 1 mm spatial resolution and 2 mm slice thickness in the axial plane, along 32 directions (b-value = 1000 s/mm^2^ repetition time 8.5 s, time to echo 61 ms), dimensions of 256 × 256 × 70, and interpolated by the MRI machine from an image with the dimensions 128 × 128 × 70. All data, initially in DICOM format, were anonymized using the GDCM toolbox [35] and processed using the FSL tool [36] for correction of eddy currents, registration of the DWI volumes, calculation of the diffusion tensor image (DTI), its eigenvalues and eigenvectors and conversion to the NIFTI format. Moreover, using the eigenvalues and eigenvectors we obtained scalar maps of the fractional anisotropy (FA), mean diffusivity (MD), axial diffusivity (AD), and radial diffusivity (RD). The mid-callosal plane, a better alternative to the midsagittal plane when dealing with corpus callosum studies, was computed directly from the DTI data using a previously proposed method that considers the highly organized fibers within the corpus callosum structure [18].

The corpus callosum was then segmented automatically using a method proposed by Rittner based on the watershed transform, a widespread technique often used for medical imaging segmentation [37]. Furthermore, the corpus callosum was parcellated (divided into parts accordingly with connected cortical regions) using the Cover scheme, a data-driven method based on DTI data within the corpus callosum segmented area [20]. It works by finding the corpus callosum medial line and using the k-means algorithm to cluster two hundred points in this line into five groups based on their FA values. The central point of each cluster is then used as seed for the watershed transform. Both mentioned segmentation and parcellation steps were performed using an open-source software called inCCsight [38], that also performed an automated quality check based on shape analysis [39].

**Figure 1:**
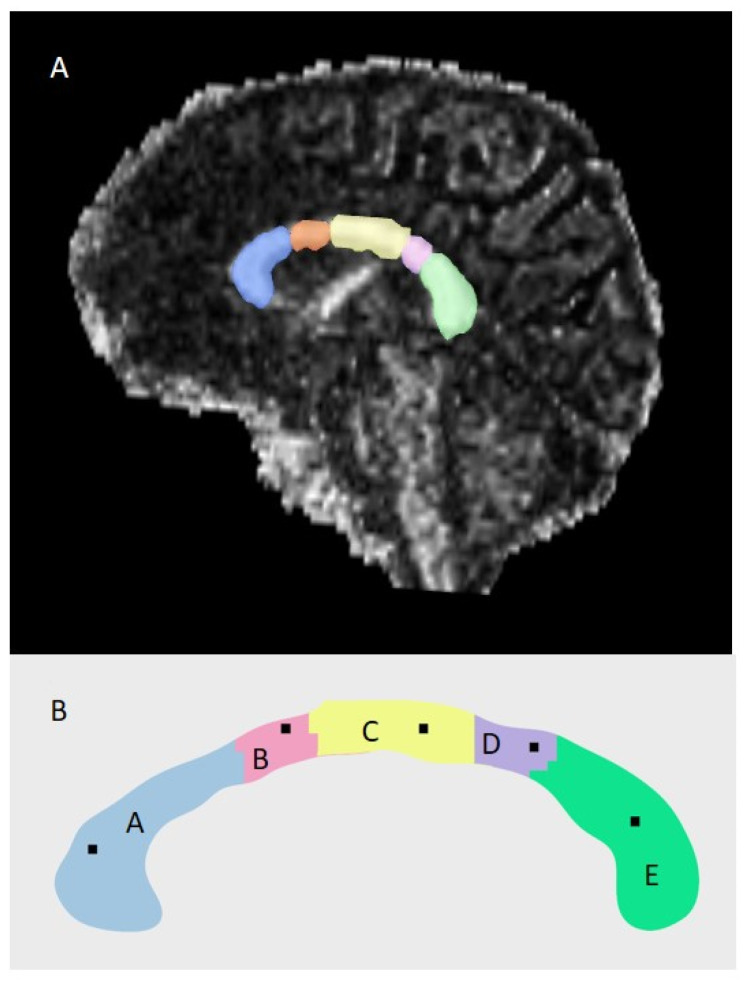
Automatic segmentation of the corpus callosum and parcellation. (**A**) Area of the corpus callosum analyzed in the study. (**B**) Description of the parcels: (A) Rostrum; (B) Genu; (C) Body; (D) Isthmus; (E) Splenium.

Using the scalar maps, we obtained the average and standard deviation of FA, MD, AD, and RD for the segmented corpus callosum and each parcel (Figure 1A,B).

### 2.8. Statistics

Descriptive statistics were performed using continuous variables presented as the mean and standard deviation. Categorical variables were compared by the chi-square test. For the comparison of DTI-based scalar maps between groups and categorical variables, an ANOVA test with a *p* value < 0.05 was used. The correlations between DTI parameters and continuous variables were explored by Spearman’s rank correlation. Bonferroni corrections for multiple comparisons was completed when indicated. Statistical analysis was performed with IBM SPSS statistics version 20, Armonk, NY: IBM Corp.

## 3. Results

We included 71 cSLE patients (mean age = 24.6 (4.6); 64 (90.1%) women) and 49 aSLE patients (mean age = 33.2 (3.72); 46 (89.8%) women) matched for sex and disease duration (Table 1). Fifty-eight controls (29.9 (3.6); 42 (72.4%) women) were included. Clinical characteristics and immunological and treatment features are shown in Table 1.

**Figure 2:**
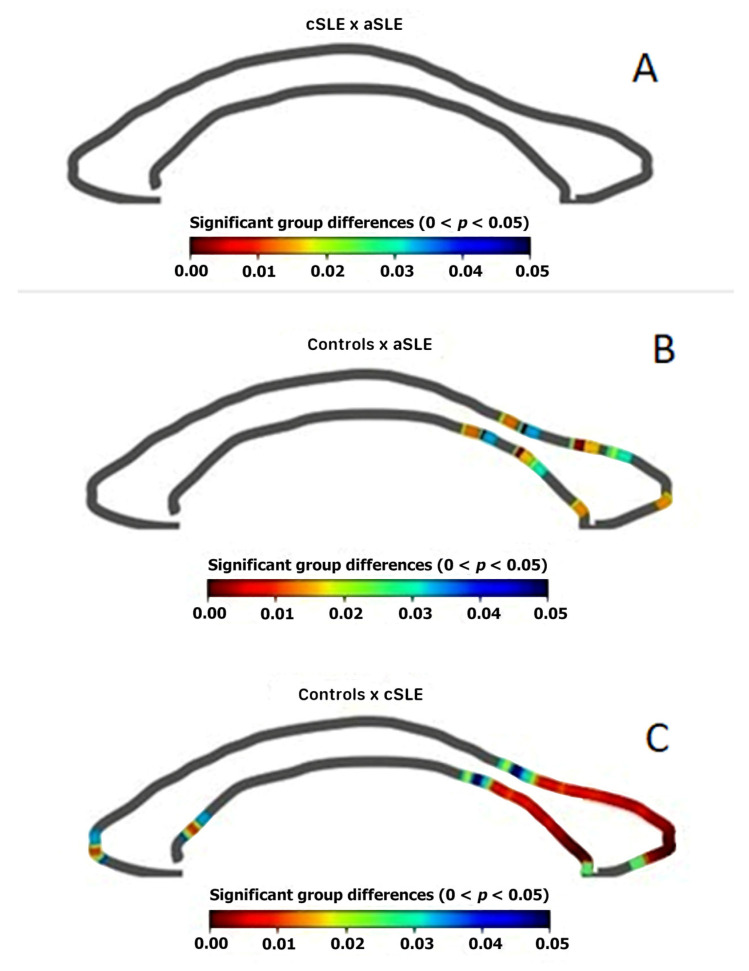
Comparison of mid-sagittal area of corpus callosum between the groups, showing a reduced area in the posterior region of the corpus callosum when comparing the controls and cSLE (**C**), the controls and aSLE (**B**), and similar areas in cSLE and aSLE (**A**).

We observed similar mid-sagittal corpus callosum areas in the cSLE and aSLE patients (Figure 2A). When we compared the aSLE patients and the controls and the cSLE patients and the controls, we observed a reduced mid-sagittal area in the posterior aspects of the corpus callosum (Figure 2B,C).

We observed significantly lower FA values in the total corpus callosum when comparing the aSLE patients to the controls, as well as higher MD and RD values (Table 2). No differences between the cSLE patients and the controls was observed.

We observed significantly lower FA values in the total corpus callosum area in cSLE patients (mean = 0.63603; SD ± 0.0665) when compared to the aSLE patints (mean = 0.677624; SD ± 0.08920; *p* = 0.002). When comparing cSLE and aSLE patients, we also observed significantly higher MD (mean = 0.00096; SD ± 0.00014 vs. mean = 0.00096; SD ± 0.00014; *p* = 0.002), RD (mean = 0.00057; SD ± 0.00015 vs. when compared with the aSLE group, mean = 0.00048; SD ± 0.00019; *p* = 0.002), and AD (mean = 0.0017; SD ± 0.00014 vs. 0.0016, SD ± 0.00011; *p* = 0.024) in cSLE (Table 2).

When we analyzed the different areas of the corpus callosum, we observed significant differences in the parcels B, C, D, and E. In all these parcels, we observed significantly lower FA and significantly higher MD, RD, and AD values in the cSLE patients when compared to the aSLE patients (Table 2).

We observed an association of lower C4 and FA, MD, RD, and D at the entire corpus callosum and with all four DTI-based scalar maps in parcels A, B, and C (*p* < 0.05). In parcel D, only associations between C4 and FA, and MD and RD (*p* < 0.05) were observed. We observed an association between DTI-based scalar maps and the presence of anticardiolipin and antiphospholipid antibodies (Table 3). No other association with immunological variables was observed.

We observed a correlation between DTI-based scalar maps and the age at disease onset (r = 0.71; *p* < 0.001) and disease duration (r = 0.481; *p* < 0.001). No association with current corticosteroid dose, hydroxychloroquine use, or antiplatelet use was observed. We observed a correlation of MoCA scores and MD values in the entire corpus callosum (r = −0.207; *p* = 0.03), as well as in all different parcels (*p* < 0.05). No other associations between DTI-based scalar maps and clinical and neuropsychiatric manifestations were observed (*p* > 0.05).

## 4. Discussion

Our study is the first to show that the corpus callosum is not uniformly affected in SLE and that microstructural changes occur predominately in the posterior region and are influenced by age of disease-onset.

In this study analyzing the mid-sagittal area of the corpus callosum, we observed significant differences in the posterior region of the corpus callosum when comparing the area between the cSLE patients and the controls and between the aSLE patients and the controls. However, no difference was observed when comparing the cSLE patients and aSLE patients. Since previous studies have analyzed the volumetric measurements of the entire corpus callosum, no data are available in the literature for comparison. Previous studies on DTI have analyzed different brain areas and observed a significant reduction in DTI-based scalar maps in SLE patients with and without neuropsychiatric manifestations and controls [40,41,42,43,44,45,46,47,48,49,50,51,52].

Our segmentation is based on a careful automated model that uses DTI parameters to differentiate corpus callosum from other structures [20]. The segmentation of the different parcels of the corpus callosum is also based on DTI parameters and has been shown to correlate with fibers received from different brain areas [12,13,14,15].

When analyzing different parcels, we observed more microstructural changes in the parcels B to D, with no changes in parcel A.

Children with SLE have a more severe disease and more disability in adulthood when compared to patients whose disease onset starts in in adulthood. The exact mechanism is not known, but inflammation and the presence of autoantibodies in the developing brain have been postulated as possible causes [3,10,11]. In this study, we have shown that cSLE patients have more microstructural damage than SLE patients with disease onset during adulthood, despite a similar disease duration. An association of DTI-based scalar maps and disease duration has been observed, indicating greater changes in longer disease duration. No significant differences in the cumulative clinical manifestations have been observed between the groups, except for a higher frequency of anxiety in aSLE patients. We did not observe any association of DTI-based scalar maps with clinical or neuropsychiatric manifestations (except cognitive impairment).

aSLE patients also had more frequently positive anti-Sm and anti-Ro antibodies. The presence of these antibodies was not associated with differences in the DTI-based scalar maps. We observed that low C4 values and the presence of anticardiolipin antibodies were associated with changes in DTI-based scalar maps. SLE patients with antiphospholipid syndrome also had more changes in DTI-based scalar maps.

No association between DTI-based scalar maps and corticosteroid use was observed. In volumetric studies, corticosteroids have been associated with cerebral atrophy [10,16,53,54].

The use of neuroimaging has significantly improved the understanding of the brain’s involvement in the disease, identifying structural and functional changes in both patients with neuropsychiatric manifestations and subclinical involvement [8,9]. However, a significant proportion of SLE patients present with normal structural MRI or unspecific findings, making care in clinical practice challenging [55,56]. The use of advanced MRI techniques and automatic segmentations allow the identification of microstructural changes. Longitudinal studies are necessary to determine if these abnormalities are risk factors for corpus callosum and brain atrophy or if they are reversible with disease remission.

Cognitive deficit is well described in the literature when related to SLE, even when neuropsychiatric manifestations are not present [57]. Cognitive deficit in SLE patients is still a challenge and several risk factors, such as neuronal autoantibodies, antiphospholipid antibodies, neuropsychiatric manifestations, and corticosteroid use have been reported as risk factors [7]. Several MRI studies, including structural and functional MRI, have identified the brain areas associated with the presence of cognitive impairment [9,10,19]. Since executive function is one of the cognitive domains more often involved in SLE, we have hypothesized that the anterior parcel of the corpus callosum would have greater microstructural abnormalities [51]. We observed a correlation of MoCA scores only with MD values in the entire corpus callosum and different parcels. No other association was observed.

This study has limitations. We were not able to retrieve the total corticosteroid dose of the patients. Longitudinal studies are necessary to determine the clinical significance of the non-uniform involvement of the corpus callosum and the use of DTI-scalar maps as biomarkers for corpus callosum atrophy.

## 5. Conclusions

We observed that the corpus callosum of adults with cSLE has more microstructural changes when compared to aSLE patients with a similar disease duration. These changes were more frequently observed in the mid and posterior area of the corpus callosum. Low complement, anticardiolipin antibodies, and cognitive impairment were associated with microstructural changes. Longitudinal studies are necessary to determine if these changes will progress to structural abnormalities or if they are reversible.

## Figures and Tables

**Table 1:** Demographic data, laboratory findings, neuropsychiatric manifestations, and treatment in cSLE, aSLE, and the controls.

Demographic Data	cSLE *N* = 71	aSLE *N* = 49	Controls *N* = 58
Sex - female	64 (90.1%)	44 (89.8%)	42 (72.4%) *
Current age (years)	24.7 (SD ± 4.6)	33.2 (SD ± 3.7) *	29.9 (SD ± 4.1)
Disease duration (years)	11.8 (SD ± 4.8)	11.3 (SD ± 4.05)	
Clinical Data			
Malar rash	38 (53.5%)	22 (44.9%)	
Discoid injury	03 (4.2%)	05 (7.0%)	
Photosensitivity	24 (33.8%) *	25 (51%)	
Oral injury	15 (21.1%) *	04 (8.2%)	
Arthritis	54 (76.1%)	35 (71.4%)	
Serositis	17 (23.9%)	17 (34.7%)	
Nephritis	35 (49.3%)	27 (55.1%)	
Hematological alteration	50 (70.4%)	35 (71.4%)	
Laboratorial Data			
↓C3	22 (31.4%)	22 (45.8%)	
↓C4	18 (25.3%)	16 (32.6%)	
ANA	69 (97.2%)	47 (96%)	
dsDNA	15 (21.4%)	19 (38.8%)	
Anti-Ro	16 (23.5%)	19 (42.2%)	
Anti-Sm	23 (33.8%)	13 (28.9%)	
Anti-La	2 (2.8%)	8 (16.3%) *	
APS	12 (16.9%)	9 (18.8%)	
Lupus anticoagulant	15 (21.1%)	8 (16.3%)	
Anticardiolipin antibodies	22 (31%)	9 (18.8%)	
Leukopenia	36 (50.7%)	29 (59.2%)	
Hemolytic anemia	9 (12.7%)	12 (24.5%)	
Thrombocytopenia	26 (36.6%)	14 (28.6%)	
NPSLE			
Overt NPSLE manifestations	53 (74.6%)	36 (73.5%)	
Anxiety	22 (31%)	26 (53.1%) *	
Depression	18 (25.4%)	14 (28.6%)	
Cognitive impairment	31 (43.7%)	16 (32.7%)	
Headache	15 (21.1%)	13 (26.5%)	
Seizure	12 (16.9%)	9 (18.4%)	
Treatment			
Corticosteroids	55 (77.5%)	42 (85.7%)	
Immunosuppressive	49 (69%)	26 (53.1%)	
Azathioprine	25 (35.2%)	16 (32.7%)	
Mycophenolate	19 (26.8%)	6 (12.2%)	
Cyclosporine	6 (8.5%)	0	
Methotrexate	0	5 (10.2%)	
Hydroxychloroquine	50 (70.4%)	36 (73.4%)	

* *p* value < 0.05 after Bonferroni correction for multiple corrections.

**Table 2:** DTI-based scalar maps in the entire corpus callosum and different parcels.

	cSLE *	aSLE *	*p* Valor *	cSLE *	Controls *	*p* Valor *	aSLE *	Controls *	*p* Valor *
**FA total**	0.63603 (±0.0665)	0.67624 (±0.08920)	**0.002**	0.63603 (±0.0665)	0.64599 (±0.0319)	0.647	0.67624 (±0.08920)	0.64599 (±0.0319)	**0.038**
**MD total**	0.00096 (±0.00014)	0.00087 (±0.00015)	**0.002**	0.00096 (±0.00014)	0.00093 (±0.00008)	0.564	0.00087 (±0.00015)	0.00093 (±0.00008)	**0.042**
**RD total**	0.00057 (±0.00015)	0.00048 (±0.00019)	**0.002**	0.00057 (±0.00015)	0.00055 (±0.0001)	0.712	0.00048 (±0.00019)	0.00055 (±0.0001)	**0.026**
**AD total**	0.0017 (±0.00014)	0.0016 (±0.00011)	**0.024**	0.0017 (±0.00014)	0.0016 (±0.0001)	0.387	0.0016 (±0.00011)	0.0016 (±0.0001)	0.383
**Parcel A**									
**FA—A**	0.66445 (±0.0744)	0.68242 (±0.1104)	0.438	0.66445 (±0.0744)	0.66942 (±0.4413)	0.932	0.68242 (±0.1104)	0.66942 (±0.4413)	0.672
**MD—A**	0.00089 (±0.0001)	0.00087 (±0.00027)	0.819	0.00089 (±0.0001)	0.00088 (±0.00007)	0.918	0.00087 (±0.00027)	0.00088 (±0.00007)	0.974
**RD—A**	0.00050 (±0.00013)	0.00047 (±0.00031)	0.737	0.00050 (±0.00013)	0.00049 (±0.00007)	0.942	0.00047 (±0.00031)	0.00049 (±0.00007)	0.910
**AD—A**	0.00168 (±0.00015)	0.00168 (±0.00022)	0.993	0.00168 (±0.00015)	0.00167 (±0.00016)	0.899	0.00168 (±0.00022)	0.00167 (±0.00016)	0.955
**Parcel B**									
**FA—B**	0.59203 (±0.10095)	0.64464 (±0.10792)	**0.005**	0.59203 (±0.10095)	0.60636 (±0.04704)	0.637	0.64464 (±0.10792)	0.60636 (±0.04704)	0.073
**MD—B**	0.00093 (±0.00025)	0.00081 (±0.00019)	**0.005**	0.00093 (±0.00025)	0.00090 (±0.00013)	0.737	0.00081 (±0.00019)	0.00090 (±0.00013)	0.054
**RD—B**	0.00060 (±0.00027)	0.00047 (±0.00023)	**0.004**	0.00060 (±0.00027)	0.00056 (±0.00022)	0.779	0.00047 (±0.00023)	0.00056 (±0.00022)	**0.039**
**AD—B**	0.00158 (±0.00025)	0.00148 (±0.00016)	**0.035**	0.00158 (±0.00025)	0.00155 (±0.00016)	0.718	0.00148 (±0.00016)	0.00155 (±0.00016)	0.212
**Parcel C**									
**FA—C**	0.58574 (±0.09617)	0.65456 (±0.09985)	**<0.001**	0.58574 (±0.09617)	0.60965 (±0.04811)	0.251	0.65456 (±0.09985)	0.60965 (±0.04811)	**0.019**
**MD—C**	0.00095 (±0.00019)	0.00083 (±0.00021)	**0.001**	0.00095 (±0.00019)	0.00091 (±0.00013)	0.376	0.00083 (±0.00021)	0.00091 (±0.00013)	0.078
**RD—C**	0.00062 (±0.00020)	0.00048 (±0.00023)	**<0.001**	0.00062 (±0.00020)	0.00058 (±0.00012)	0.402	0.00048 (±0.00023)	0.00058 (±0.00012)	**0.027**
**AD—C**	0.00161 (±0.00019)	0.00153 (±0.00019)	0.126	0.00161 (±0.00019)	0.00157 (±0.00018)	0.493	0.00153 (±0.00019)	0.00157 (±0.00018)	0.681
**Parcel D**									
**FA—D**	0.58105 (±0.09756)	0.63433 (±0.10684)	**0.006**	0.58105 (±0.09756)	0.59873 (±0.06512)	0.518	0.63433 (±0.10684)	0.59873 (±0.06512)	0.112
**MD—D**	0.00108 (±0.00030)	0.00090 (±0.00016)	**<0.001**	0.00108 (±0.00030)	0.00102 (±0.00014)	0.340	0.00090 (±0.00016)	0.00102 (±0.00014)	**0.020**
**RD—D**	0.00072 (±0.00032)	0.00054 (±0.00020)	**<0.001**	0.00072 (±0.00032)	0.00067 (±0.00015)	0.463	0.00054 (±0.00020)	0.00067 (±0.00015)	**0.020**
**AD—D**	0.00179 (±0.00028)	0.00164 (±0.00015)	**<0.001**	0.00179 (±0.00028)	0.00173 (±0.00015)	0.221	0.00164 (±0.00015)	0.00173 (±0.00015)	0.068
**Parcel E**									
**FA—E**	0.68225 (±0.06640)	0.72460 (±0.04514)	**<0.001**	0.68225 (±0.06640)	0.69112 (±0.04791)	0.638	0.72460 (±0.04514)	0.69112 (±0.04791)	**0.006**
**MD—E**	0.00099 (±0.00013)	0.00090 (±0.00007)	**<0.001**	0.00099 (±0.00013)	0.00096 (±0.00010)	0.341	0.00090 (±0.00007)	0.00096 (±0.00010)	**0.005**
**RD—E**	0.00055 (±0.00013)	0.00044 (±0.00008)	**<0.001**	0.00055 (±0.00013)	0.00053 (±0.00010)	0.600	0.00044 (±0.00008)	0.00053 (±0.00010)	**<0.001**
**AD—E**	0.00188 (±0.00019)	0.00180 (±0.00014)	**0.042**	0.00188 (±0.00019)	0.00184 (±0.00016)	0309	0.00180 (±0.00014)	0.00184 (±0.00016)	0.582

* Values expressed in mm^2^/s. Values expressed as mean and ± standard deviation. Statistical test: ANOVA.

**Table 3:** DTI scalar maps and association with reduced C4, the presence of anticardiolipin antibodies, and antiphospholipid syndrome. Statistical test: ANOVA.

DTI Scalar Maps	Reduced C4	Positive Anticardiolipin Antibodies	Antiphospholipid Syndrome
**FA total**	0.016	0.003	0.065
**MD total**	0.003	0.019	0.102
**RD total**	0.003	0.008	0.093
**AD total**	0.035	0.334	0.259
**FA—A**	0.059	0.000	0.001
**MD—A**	0.004	0.005	0.007
**RD—A**	0.005	0.001	0.002
**AD—A**	0.025	0.290	0.196
**FA—B**	0.026	0.017	0.058
**MD—B**	0.161	0.242	0.283
**RD—B**	0.086	0.122	0.196
**AD—B**	0.500	0.893	0.681
**FA—C**	0.037	0.222	0.519
**MD—C**	0.006	0.033	0.208
**RD—C**	0.007	0.051	0.292
**AD—C**	0.037	0.039	0.146
**FA—D**	0.006	0.036	0.073
**MD—D**	0.007	0.039	0.123
**RD—D**	0.022	0.028	0.081
**AD—D**	0.002	0.146	0.393
**FA—E**	0.578	0.058	0.958
**MD—E**	0.159	0.048	0.461
**RD—E**	0.159	0.034	0.841
**AD—E**	0.159	0.305	0.213

## Data Availability

Data are available upon specific request.
